# Significance of ligand interactions involving Hop2-Mnd1 and the RAD51 and DMC1 recombinases in homologous DNA repair and XX ovarian dysgenesis

**DOI:** 10.1093/nar/gkv259

**Published:** 2015-03-27

**Authors:** Weixing Zhao, Patrick Sung

**Affiliations:** Department of Molecular Biophysics and Biochemistry, Yale University School of Medicine, New Haven, CT 06520, USA

## Abstract

The evolutionarily conserved Hop2-Mnd1 complex is a key cofactor for the meiosis-specific recombinase Dmc1. However, emerging evidence has revealed that Hop2-Mnd1 is expressed in somatic tissues, primary human fibroblasts and cell lines, and that it functions in conjunction with the Rad51 recombinase to repair damaged telomeres via the alternate lengthening of telomeres mechanism. Here, we reveal how distinct DNA-binding activities of Hop2-Mnd1 mediate the stabilization of the RAD51-ssDNA presynaptic filament or stimulate the homologous DNA pairing reaction. We have also endeavored to define the interface that governs the assembly of the higher order complex of Hop2-Mnd1 with RAD51. Unexpectedly, we find that ATP enhances the interaction between Hop2-Mnd1 and RAD51, and that both Hop2 and Mnd1 are involved in RAD51 interaction via their C-terminal regions. Importantly, mutations introduced into these Hop2 and Mnd1 domains, including the *HOP2* p.del201Glu mutation present in a patient of XX ovarian dysgenesis, diminish the association and functional synergy of Hop2-Mnd1 with both RAD51 and DMC1. Our findings help delineate the intricate manner in which Hop2-Mnd1 engages and functions with RAD51 and DMC1 in mammalian cells and speak to the possible cause of XX ovarian dysgenesis.

## INTRODUCTION

Homologous recombination (HR) repairs damaged chromosome and, as such, serves an important role in maintaining genome stability (1). In addition, HR-mediated pairing of homologous chromosomes is critical for their proper disjunction in the first meiotic division and the generation of genetically diverse gametes (2). Accordingly, dysfunction in HR often predisposes the affected individuals to cancer, or could lead to infertility, birth defects, and developmental problems including Down (trisomy 21), Turner (monosomy for X) and Klinefelter (XXY male) syndromes (2).

The HR reaction in eukaryotic cells is mediated by either Rad51 or Dmc1, which both possess a recombinase activity capable of catalyzing homologous DNA pairing and strand exchange (1). While Rad51 is present in both mitotic and meiotic cells, Dmc1 is expressed only in meiosis (3,4). During catalysis, Rad51 and Dmc1 associate with ssDNA derived from the nucleolytic processing of a primary lesion, e.g. a DNA double-stranded break, to form a right-handed helical nucleoprotein filament, commonly referred to as the presynaptic filament. With the aid of accessory factors, the presynaptic filament engages a duplex DNA molecule and searches for a homologous sequence in the latter. Upon the location of homology, the presynaptic filament invades the duplex target to form a nascent heteroduplex DNA joint, the D-loop, the length of which is extended by DNA strand exchange and DNA synthesis. Resolution of the D-loop intermediate occurs via one of several mechanistically distinct pathways, with the potential of generating either crossover or non-crossover DNA recombinants (5).

The heterodimeric Hop2-Mnd1 complex is a conserved recombinase cofactor (6–10) that stabilizes the presynaptic filament and synergizes with the latter in the capture of duplex DNA to assemble the synaptic complex, in which the recombining DNA partners are aligned in homologous registry, with limited base switching having occurred between the DNA partners (11–14). Moreover, Hop2-Mnd1 regulates ATP and DNA binding by RAD51 in a manner that is beneficial for homologous DNA pairing and strand exchange (14). While Hop2-Mnd1 from the budding yeast *Saccharomyces cerevisiae* is expressed in meiotic cells exclusively and functions only with Dmc1 (6,15), emerging biochemical and genetic evidence has revealed a mitotic role of Hop2-Mnd1 via interaction and functional synergy with Rad51 in higher eukaryotes (16,17). For instance, in *Arabidopsis thaliana*, the expression of Hop2 and Mnd1 is induced by γ-rays, and *mnd1* mutant plants are impaired in vegetative growth after exposure to γ−irradiation (16). In a recent study, Hop2-Mnd1 was shown to be expressed in primary human fibroblasts and cell lines (17), and cell biological results were furnished to show that Hop2-Mnd1 functions with RAD51 in recombination events that lead to telomere lengthening. Importantly, mutations in Hop2 have been found in early onset familial breast and ovarian cancer patients (18,19), and a single amino acid deletion (Glu201 del) has been tied to XX ovarian dysgenesis that is characterized by streak ovaries (20).

Herein, we provide evidence that the ssDNA-binding activity of the Hop2-Mnd1 complex helps stabilize the RAD51 presynaptic filament and that its dsDNA binding attribute functionally synergizes with the presynaptic filament to assemble the synaptic complex. We have also strived to delineate the interaction interface in Hop2-Mnd1 that mediates its interaction with RAD51. In this regard, results are presented to (i) show an unexpected level of complexity in how Hop2-Mnd1 associates with RAD51 through contacts on both subunits, (ii) reveal that the same Hop2-Mnd1 domains are involved in DMC1 interaction, (iii) ascertain the relevance of protein complex formation in the RAD51- and DMC1-mediated homologous DNA pairing and strand exchange reaction and (iv) suggest a linkage of ensembles of Hop2-Mnd1 with RAD51 and DMC1 to XX ovarian dysgenesis.

## MATERIALS AND METHODS

### Plasmids

The protein expression vectors pRSF-(His)_6_-Hop2-Mnd1 (encoding (His)_6_-tagged Hop2 and untagged Mnd1), pRSF-(His)_6_-Hop2-MBP-Mnd1 (encoding N-terminally (His)_6_-tagged Hop2 and N-terminally MBP-tagged Mnd1) and their truncation mutants were used in this study, as described (12). Construction of mutants was done using the Quickchange mutagenesis kit (Stratagene).

### Protein purification

Mouse Hop2-Mnd1, human RAD51 and their mutant derivatives were expressed in *Escherichia coli* and purified as described (12,21). Human DMC1 was purified from insect cells infected with a recombinant DMC1 baculovirus, as described (22).

### Affinity pulldown assay

RAD51 (8 μM) was incubated with 5 μM of wild type or mutant (His)_6_-Hop2-Mnd1 at 4°C for 30 min in 30 μl buffer A (25 mM Tris-HCl, pH 7.5, 10% glycerol, 0.5 mM EDTA, 0.01% Igepal CA-630 (Sigma), 1 mM 2-mercaptoethanol, 10 mM imidazole and the indicated concentration of KCl) with or without 2 mM MgCl_2_ and 2 mM ATP. After mixing with 12 μl Ni^2+^-NTA resin (Qiagen) at 4°C for 30 min, the resin was washed three times with 100 μl buffer A and then treated with 20 μl 2% SDS to elute proteins. The supernatant (S), final wash (W) and SDS eluate (E) fractions, 8 μl each, were analyzed by sodium dodecyl sulphate-polyacrylamide gel electrophoresis (SDS-PAGE). For experiments with MBP-tagged Hop2-Mnd1, the same procedure was followed except that imidazole was omitted from the buffer, and that 12 μl of amylose resin (New England Biolabs) was used to capture protein complexes.

### DNA binding assay

DNA binding was done with ^32^P-labeled 80-mer ssDNA and dsDNA substrates with analysis in polyacrylamide gels exactly as described (12).

### D-loop reaction

This was conducted as described (12,23). Briefly, ^32^P-labeled 90-mer oligonucleotide (2.4 μM nucleotides) was preincubated with RAD51 or DMC1 (1 μM) for 5 min at 37°C in the reaction buffer (25 mM Tris-HCl, pH 7.5, 60 mM KCl, 1 mM DTT, 1 mM (for RAD51) or 5 mM (for DMC1) MgCl_2_, 2 mM ATP, and 100 μg/ml BSA). This was followed by the incorporation of Hop2-Mnd1 or mutant and a 5-min incubation at 37°C. The D-loop reaction was initiated by adding pBluescript SK replicative form I DNA (37 μM base pairs) and incubated at 37°C for 7 min. The molar ratio of the 90-mer to pBluescript plasmid in the reactions was 2.1 to 1. After electrophoresis in a 1% agarose gel, phosphorimaging analysis was carried out to visualize and quantify the radiolabeled DNA species (23). Where indicated, 1 mM CaCl_2_ was included in the reaction buffer or AMP-PNP was used instead of ATP.

### Recombinase turnover from ssDNA

The RAD51-ssDNA presynaptic filament stabilization test was conducted using magentic beads containing ssDNA exactly as described (13; see Figure 2a).

**Figure 1.**
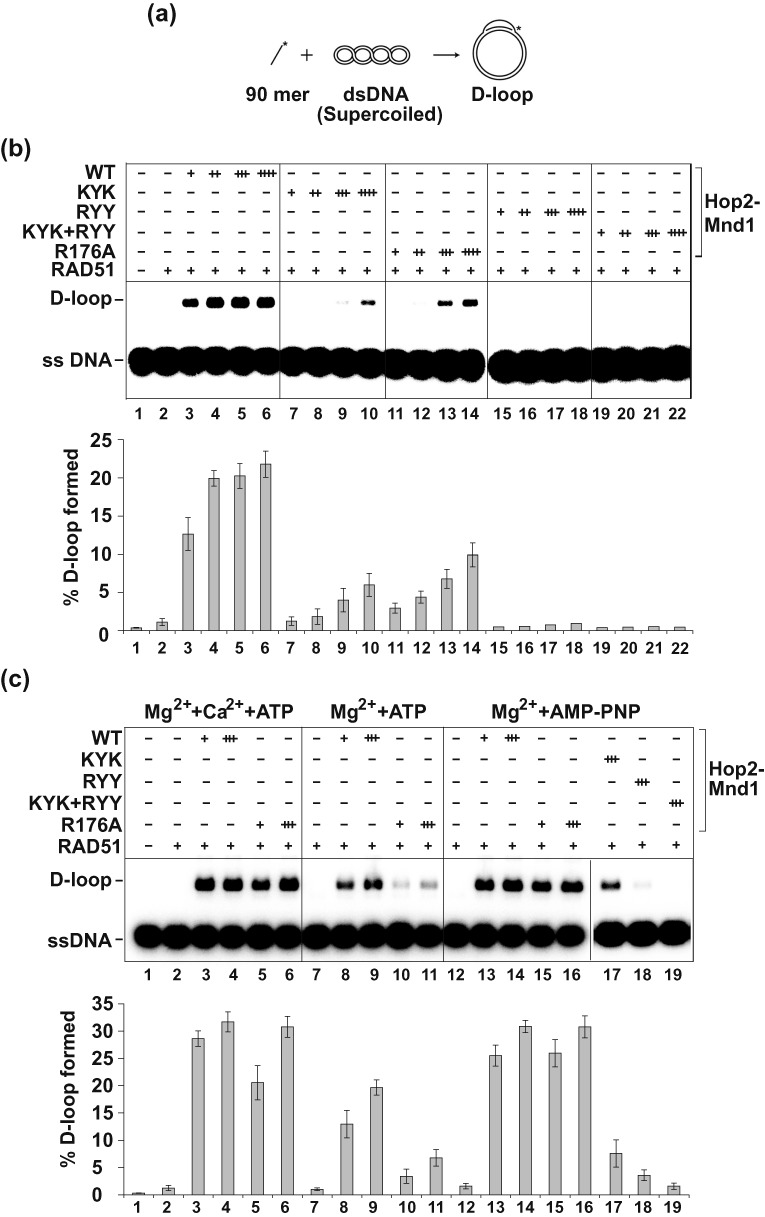
Examination of DNA binding mutants of Hop2-Mnd1 in the D-loop reaction catalyzed by RAD51. **(a)** Schematic of the D-loop reaction (23). **(b)** Wild type (WT) and mutant variants of the Hop2-Mnd1 complex (60 nM, 120 nM, 180 nM or 240 nM) were tested for their ability to promote D-loop formation with ATP as nucleotide cofactor. **(c)** WT and mutant variants of the Hop2-Mnd1 complex (60 nM or 180 nM) were tested for their ability to promote D-loop formation under three different sets of conditions: Ca^2+^+Mg^2+^+ATP (left panel), Mg^2+^+ATP (middle panel) and Mg^2+^+AMP-PNP (right panel). In (b) and (c), the mean values ± s.d. from three independent experiments were plotted.

**Figure 2.**
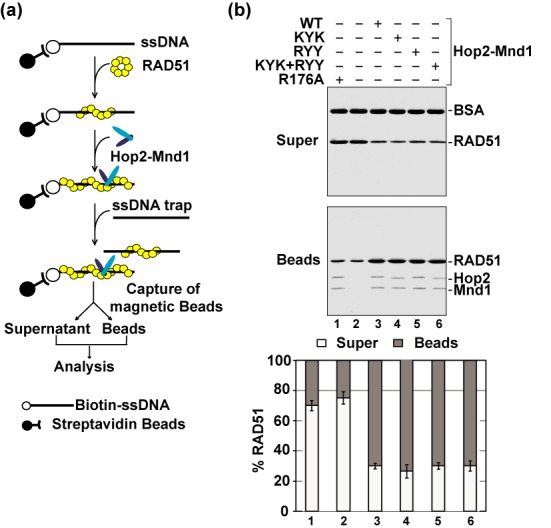
Role of the Hop2 C-terminal DNA binding domain in RAD51 presynaptic filament stabilization. **(a)** Schematic of the presynaptic filament stabilization assay (13). **(b)** Wild type (WT) and mutant variants of the Hop2–Mnd1 complex were tested for their ability to stabilize the RAD51 presynaptic filament. The mean values ± s.d. from three independent experiments were plotted.

### Duplex DNA capture

**Figure 3.**
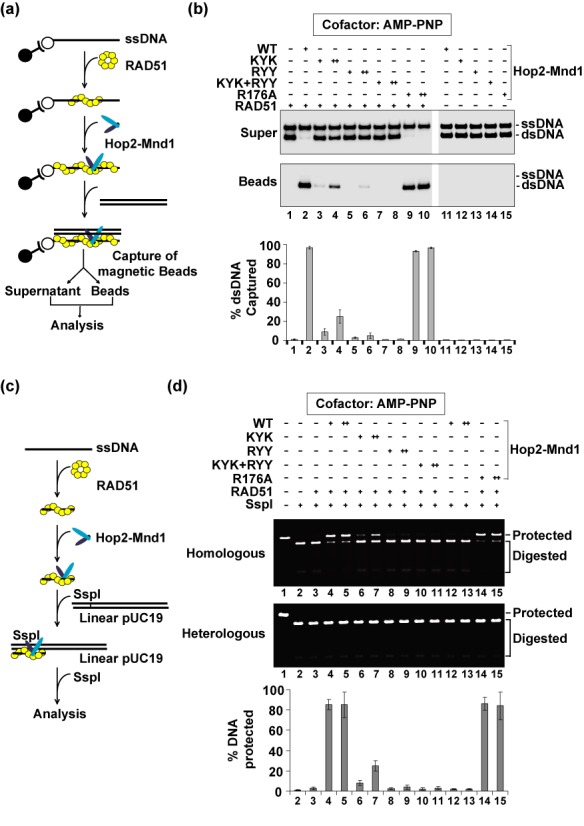
Role of the N-terminal DNA binding domains of Hop2-Mnd1 in duplex DNA capture and synaptic complex assembly. **(a)** Schematic of the duplex capture assay (13). **(b)** Wild type (WT) and mutant variants of the Hop2-Mnd1 complex (0.6 or 1.2 μM) were tested for their ability to promote duplex capture with AMP-PNP as nucleotide cofactor. **(c)** Schematic of the restriction enzyme protection assay to examine synaptic complex formation (12,25). **(d)** WT and mutant variants of the Hop2-Mnd1 complex (0.48 or 0.96 μM) were tested for their ability to promote synaptic complex formation with AMP-PNP as nucleotide cofactor. In (b) and (d), the mean values ± s.d. from three independent experiments were plotted.

RAD51 (2.7 μM) and biotinylated 83-mer oligo dT (12 μM nucleotides) were used for examining duplex DNA capture with Hop2-Mnd1 and its mutants exactly as described (13; see Figure 3a). Either ATP or AMP-PNP was used as the nucleotide cofactor as indicated.

### Synaptic complex formation

RAD51 (4 μM) and 60-mer ssDNA (12 μM nucleotide) were used for testing synaptic complex formation with Hop2-Mnd1 or its mutants exactly as described (11; see Figure 3c). AMP-PNP was used as the nucleotide cofactor.

### Yeast two- and three-hybrid analyses

pBridge^TM^ and pGADT7 vector (Clontech) were used to set up the three-hybrid system to investigate ternary complex formation among Hop2, Mnd1, RAD51 and DMC1 in the reporter yeast strain AH109 (Clontech). Briefly, pBridge^TM^ allows expression of a DNA binding (BD) fusion protein and a third protein (for ‘three-hybrid’ analysis), while the activation domain (AD) vector pGADT7 provides the AD fusion protein. When BD and AD fusions interact with AH109, the DNA-BD and AD are brought into proximity and activate transcription of the *HIS3* and *ADE2* reporter genes. First, pBridge^TM^ (carrying the *TRP1* gene) and pGADT7 (carrying the *LEU2* gene) were introduced into AH109 cells by selection on Leu-Trp- medium. Then, Colonies were resuspended in liquid, serially diluted and streaked on Leu-Trp-His-(medium stringency) and Leu-Trp-His-Ade- (high stringency) media.

## RESULTS

### Role of the Hop2 C-terminal DNA binding domain in presynaptic filament stabilization

Focusing on the mouse Hop2-Mnd1 complex, our published study has revealed three distinct DNA binding domains within the complex, with two of these domains having specificity for dsDNA and being located within the N-terminal region of Hop2 and Mnd1, while the C-terminal region of Hop2 possesses another such domain with specificity for ssDNA (12). Here, using the four available DNA binding mutant variants of mouse Hop2-Mnd1 (Table 1; (12)), we investigated how the DNA binding attributes of Hop2-Mnd1 affect the D-loop reaction mediated by human RAD51.

**Table 1.** Summary of the properties of Hop2-Mnd1 and its mutants

Hop2-Mnd1 and its mutants	dsDNA binding	ssDNA binding	RAD51 interaction	DMC1 interaction	Reference
Hop2-Mnd1	+	+	+	+	12
Hop2 (K63A/Y65A/K67A)-Mnd1	-	-	+	+	12
Hop2-Mnd1 (R64A/Y70A/Y71A)	-	+	+	+	12
Hop2 (R176A)-Mnd1	+	-	+	+	12
Hop2-Mnd1 (KYK+RYY)	-	-	+	+	12
Hop2^1-143^-Mnd1	+	-	-	nd	12
Hop2^126-217^-Mnd1	-	-	+	nd	12
Hop2-Mnd1^1-113^	+	+	-	nd	12
Hop2-Mnd1^100-205^	-	+	+	nd	12
Hop2-Mnd1^1-185^	+	+	-	-	This study
Hop2^1-190^-Mnd1	+	+	-	-	This study
Hop2-Mnd1 (F195A)	+	+	-	-	This study
Hop2-Mnd1 (I197Q)	+	+	-	-	This study
Hop2-Mnd1 (F201A)	+	+	-	-	This study
Hop2-Mnd1 (F195A/I197Q/F201A)	+	+	-	-	This study
Hop2 (K193A/K195A/K196A)-Mnd1	nd	nd	+*	nd	This study
Hop2 (F198A/F199A)-Mnd1	+	+	-	-	This study
Hop2 (G203N)-Mnd1	+	+	+	+	This study
Hop2 (V202Q/I204Q)-Mnd1	+	+	-	-	This study
Hop2 (V212Q)-Mnd1	nd	nd	+*	nd	This study
Hop2 (L213Q/L214Q)-Mnd1	nd	nd	+*	nd	This study
Hop2 (ΔE201)-Mnd1	+	+	-	-	This study

Note: ‘+’ denotes wild-type activity; ‘-’ denotes an impairment of activity; nd = not determined. The asterisk (*) denotes yeast three-hybrid interaction.

First, by affinity pulldown, we determined that the mutant Hop2-Mnd1 complexes are proficient in RAD51 interaction (summarized in Table 1). Importantly, we found that these mutant complexes are, to a varying degree, impaired for the ability to stimulate the D-loop reaction (Figure 1a and b), with the strongest defect being seen with the Hop2-Mnd1-RYY and Hop2-KYK-Mnd1-RYY mutants. Interestingly, and as we previously showed for DMC1 (12), the Hop2-R176A-Mnd1 mutant, but none of the remaining mutants, is as proficient as the wild-type complex in the enhancement of D-loop formation when either Ca^2+^ ion is included in the reaction buffer or when AMP-PNP is used as the nucleotide cofactor (Figure 1c), regimens that lead to stabilization of the RAD51 presynaptic filament (23,24). We next examined the Hop2-Mnd1 mutants in a biochemical assay designed to test the effect of recombinase cofactors on presynaptic filament stability (Figure 2a, (13)). The results revealed that while the Hop2-KYK-Mnd1, Hop2-Mnd1-RYY and Hop2-KYK/Mnd1-RYY mutants retain the ability to stabilize the presynaptic filament, the Hop2-R176A-Mnd1 mutant lacks this attribute (Figure 2b).

### Role of the Hop2-Mnd1 N-terminal DNA binding domains in synaptic complex assembly

We employed two distinct assay systems—duplex DNA capture (Figure 3a) and protection against a restriction endonuclease (Figure 3c) (12,13,25)—to test the ability of the Hop2–Mnd1 mutant complexes to work in conjunction with the RAD51 presynaptic filament in assembling the synaptic complex. In these experiments, either ATP or AMP-PNP was used as the nucleotide cofactor. The results showed that while the Hop2-KYK-Mnd1, Hop2-Mnd1-RYY and Hop2-KYK/Mnd1-RYY mutants are impaired in duplex capture and protection against DNA digestion regardless of the nucleotide used, Hop2-R176A-Mnd1 mutant is deficient in these reactions only in the presence of ATP (Figure 3b and d and Supplementary Figure S1).

Taken together, the results presented herein and in the preceding section revealed that the ssDNA-binding activity of Hop2 is needed for RAD51 presynaptic filament stabilization, whereas efficient synaptic complex assembly is reliant on the two dsDNA binding domains residing in the N-termini of Hop2 and Mnd1.

### Enhancement of interaction between RAD51 and Hop2-Mnd1 by ATP

**Figure 4.**
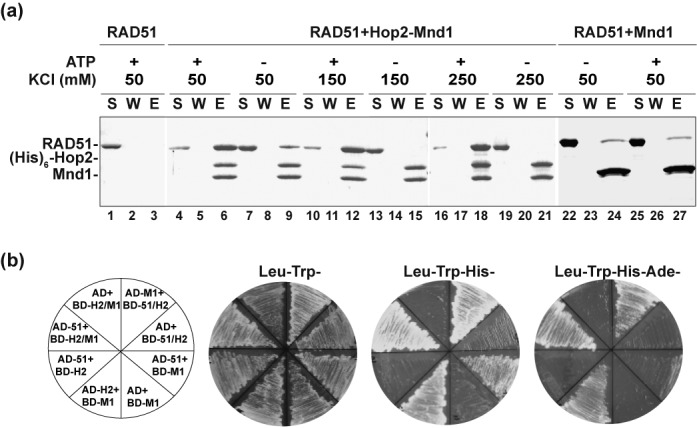
The robust interaction of Hop2-Mnd1 with RAD51 is induced by ATP and requires both subunits of the Hop2-Mnd1 complex. **(a)** Affinity pulldown to test for the interaction between RAD51 and (His)_6_-tagged Hop2-Mnd1 or (His)_6_-tagged Mnd1 using Ni^2+^-NTA resin to capture protein complexes. The reactions contained the indicated concentration of KCl and with or without ATP. The supernatant (S) containing unbound proteins, the wash (W) and the eluate (E) fractions were analyzed by SDS-PAGE and Coomassie Blue staining. **(b)** Yeast three-hybrid analysis to test for the interaction of RAD51 with Hop2, Mnd1 and Hop2-Mnd1. Abbreviations: H2, Hop2; M1, Mnd1; 51, RAD51.

The association of RAD51 with Hop2-Mnd1 is salt sensitive, such that increasing the KCl concentration from 50 to 150 mM strongly diminishes the level of protein complex (Figure 4a; (13)). Since ATP is needed for recombinase activity and appears to induce a conformational change in RAD51 (26), we investigated whether it may regulate complex formation between Hop2-Mnd1 and RAD51. Interestingly, we found a strong enhancement of the association of RAD51 with Hop2-Mnd1 by ATP, and that the protein complex so made is stable to 250 mM KCl (Figure 4a). We also showed that RAD51-K133A, a mutant variant not expected to bind ATP, remains unresponsive to ATP in Hop2-Mnd1 interaction (Supplementary Figure S2a). These findings thus revealed that ATP promotes the interaction of RAD51 with Hop2-Mnd1. Additional results further showed that (i) dATP and AMP-PNP also stimulate interaction of RAD51 with Hop2-Mnd1, but ATP-γS, ADP and AMP are ineffective in this regard (Supplementary Figure S2b) and (ii) neither *S. cerevisiae* Rad51 nor *E. coli* RecA interacts with Hop2-Mnd1 even in the presence of ATP (Supplementary Figure S2c). Finally, even though Hop2-Mnd1 complex also physically interacts and functions with DMC1, complex formation between them is insensitive to ATP (data not shown).

### Involvement of both Hop2 and Mnd1 in RAD51 interaction

Even though purified Mnd1 alone can bind RAD51 (13), formation of the protein complex is ATP insensitive (Figure 4a). Little or no interaction between purified Hop2 and RAD51 occurs with or without ATP (data not shown). Based on these observations, we speculated that Hop2 might also contribute to RAD51 interaction within the context of the Hop2-Mnd1 complex.

We employed the yeast two- and three-hybrid assays to further explore the nature of protein–protein interactions between Hop2-Mnd1 and RAD51. Association of the bait and prey in the system permits Gal4-dependent transcription of reporter genes, which then enables the tester yeast strain to acquire adenine/histidine prototrophy. Consistent with the affinity pulldown data (Figure 4a), an association with RAD51 was found for Mnd1, while no interaction could be detected between Hop2 and RAD51 (Figure 4b). Importantly, a strong interaction with RAD51 was observed only when RAD51 was co-expressed with both Hop2 and Mnd1 but not with either of the latter two proteins alone (Figure 4b). Together, the results in this and the preceding sections provided the first evidence that the robust association of RAD51 with Hop2-Mnd1 requires, in addition to the known interaction with Mnd1, the participation of Hop2.

### RAD51 interaction domains in the C-termini of Hop2 and Mnd1

Following up on the yeast two- and three-hybrid results, we tested several available truncation mutants (Table 1) (12) for their ability to interact with RAD51 in affinity pulldown experiments. The results revealed that Hop21-143-Mnd1 and Hop2-Mnd1^1-113^, deleted for the C-terminal region of either Hop2 or Mnd1 (Supplementary Figure S3a), respectively, are strongly impaired for RAD51 interaction (Supplementary Figure S3b). In contrast, deletion of the N-terminal region of either Hop2 or Mnd1, as in Hop2^126-^^217^-Mnd1 and Hop2-Mnd1^100-205^, respectively, was found to have little or no affect on RAD51 association (Supplementary Figure S3b and Table 1). Based on the above information, we proceeded to delineate the RAD51 interaction regions in Hop2-Mnd1 further. Importantly, Hop2-Mnd1 mutants, namely, Hop2^1-190^-Mnd1 and Hop2-Mnd1^1-185^, that bear a shorter deletion in their respective C-terminal region (27 residues of Hop2 or 20 residues of Mnd1) are also strongly impaired for RAD51 association (Supplementary Figure S3c and Table 1). Importantly, the aforementioned Hop2 and Mnd1 C-terminal truncation mutations strongly impair the ability of the Hop2-Mnd1 complex to enhance RAD51-mediated D-loop formation (data not shown). Together, the results presented herein indicate that the C-termini of Hop2 and Mnd1 are needed for maximal affinity of the Hop2-Mnd1 complex for RAD51.

### Hop2 and Mnd1 point mutants defective in RAD51 interaction

**Figure 5.**
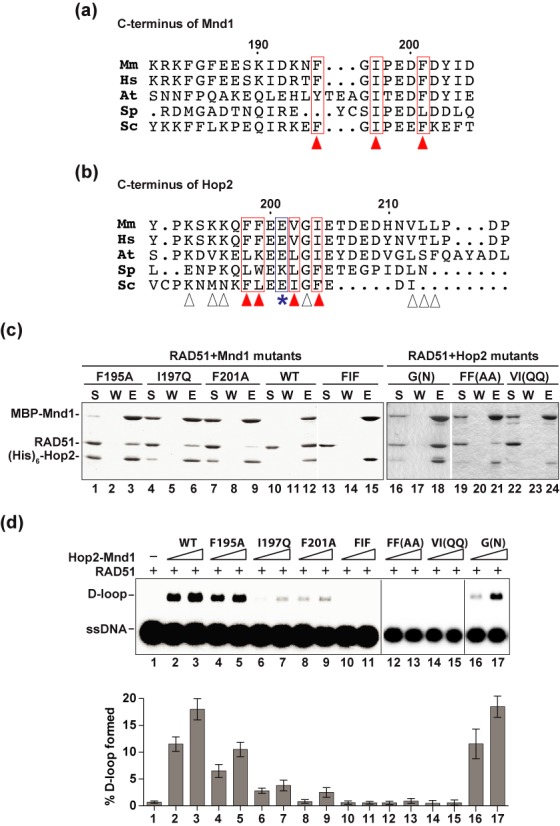
Hop2 and Mnd1 mutants defective in interaction with RAD51. **(a)** Alignment of the C-termini of Mnd1 orthologs. The arrowheads highlight the residues selected as mutagenesis targets. **(b)** Alignment of the C-termini of Hop2 orthologs. The arrowheads highlight the residues picked as mutagenesis targets (red arrowheads denote those residues that impair recombinase interaction when mutated; white arrowheads denote those mutations that do not affect recombinase interaction when mutated). The E201 residue that is deleted in a case of XX-GD is denoted by the asterisk. Note that there is only limited overall sequence similarity between the yeast and mammalian Hop2 and Mnd1 species in their C-terminal region (a and b), which could explain why the yeast Hop2–Mnd1 complex does not interact with Rad51. **(c)** Pulldown assay to test for the interaction of RAD51 with MBP-tagged Hop2-Mnd1 and mutants using amylose resin to capture protein complexes. Analysis was conducted as in Figure 4a. **(d)** Wild type (WT) and mutant variants of the Hop2-Mnd1 complex (90 or 180 nM) were tested for their ability to promote D-loop formation by RAD51. The mean values ± s.d. from three independent experiments were plotted. Abbreviations in (a) and (b): Mm, *Mus musculus*; Hs, *Homo sapiens*; At, *Arabidopsis thaliana*; Sp, *Schizosaccharomyces pombe*; Sc, *Saccharomyces cerevisiae*.

To ascertain the functional relevance of the higher order ensemble of RAD51 and Hop2-Mnd1, we strived to isolate point mutants of Hop2 and Mnd1 that affect RAD51 interaction but have no impact on the formation of the Hop2-Mnd1 complex or on its DNA-binding activity. For this purpose, we aligned the C-terminal regions of Hop2 and Mnd1 orthologs, so as to identify conserved residues as the mutagenesis targets (Figure 5a and b).

For Mnd1, we changed F195, I197 and F201 (Figure 5a), singly or simultaneously to alanine or glutamine, and tested the mutants within the context of the Hop2-Mnd1 complex for RAD51 interaction (Figure 5c). Importantly, all three point mutations (i.e. F195A, I197Q and F201A) significantly impair the RAD51 interaction capability of Hop2-Mnd1, while the compound mutant (F195A/I197Q/F201A) is devoid of such capability (Figure 5c and Table 1). Accordingly, those mutants are defective in stimulating RAD51-mediated D-loop formation to degrees (Figure 5d) that reflect the severity of their RAD51 interaction defects (Figure 5c). We note that none of the aforementioned Mnd1 mutations affects the DNA-binding activity of Hop2-Mnd1 (summarized in Table 1).

As shown in Figure 5b, the C-terminus of Hop2 is not as conserved as that of Mnd1 among orthologs. We made many mutants of Hop2 that harbor changes in this region and first tested them in the yeast three-hybrid system for RAD51 interaction. The results showed that the Hop2 K193A/K195A/K196A, L212Q/L213Q, G203N and V212Q mutations have little or no impact on the interaction of Hop2-Mnd1 with RAD51 (Supplementary Figure S4 and Table 1). However, the F198A/F199A and V202Q/I204Q mutations render Hop2-Mnd1 defective in RAD51 interaction (Supplementary Figure S4 and Table 1). We proceeded to express and purify Hop2-Mnd1 complexes that harbor the F198A/F199A, V202Q/I204Q or G203N mutation and tested them for RAD51 binding by biochemical pull-down. Consistent with the yeast three-hybrid results, we found that the F198A/F199A, V202Q/I204Q mutants are severely impaired for RAD51 interaction (Figure 5c and Table 1) and, importantly, neither of these mutants is able to enhance the D-loop reaction. In contrast, the G203N mutant is just as proficient as wild-type Hop2-Mnd1 in RAD51 interaction and the enhancement of D-loop formation (Figure 5d). The three Hop2 mutations have no negative impact on the DNA-binding activity of Hop2-Mnd1 (summarized in Table 1).

### Interactions with DMC1 and RAD51 occur through common Hop2-Mnd1 domains

**Figure 6.**
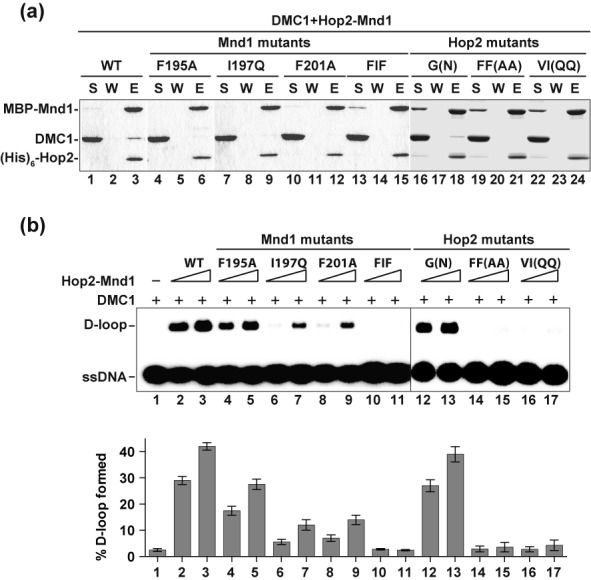
Impairment of DMC1 interactions by Hop2 and Mnd1 mutations. **(a)** Pulldown assay to test for the interaction of DMC1 with MBP-tagged Hop2-Mnd1 and mutants using amylose resin to capture protein complexes. Analysis was conducted as in Figure 4a. **(b)** Wild type (WT) and mutant variants of the Hop2-Mnd1 complex (90 or 180 nM) were tested for their ability to promote D-loop formation by DMC1. The mean values ± s.d. from three independent experiments were plotted.

Previously published work has shown that the C-terminus of Mnd1 is indispensable for interaction with human DMC1 (12), so it was of considerable interest to ask whether Hop2-Mnd1 employs the RAD51 interaction interfaces to associate with DMC1 as well. As shown in Figure 6, we found that the interaction between Hop2-Mnd1 with DMC1 is also impaired by the same Hop2 (F198A/F199A and V202Q/I204Q) and Mnd1 (F195A, I197Q, F201A and F195A/I197Q/F201A) mutations that affect RAD51 interaction, but not by the Hop2 G203N mutation that has no impact on RAD51 association (Figure 6a and Table 1). Accordingly, with the exception of Hop2 G203N, the aforementioned mutations either diminish (Mnd1 F195A, I197Q and F201A) or ablate (Hop2 F198A/F199A and V202Q/I204Q; Mnd1 F195A/I197Q/F201A) the efficacy of Hop2-Mnd1 in the D-loop reaction (Figure 6b). Together, the results provide evidence that Hop2-Mnd1 employs the same protein domains to associate with DMC1 and RAD51.

### Linkage of Hop2-Mnd1-recombinase complexes to XX ovarian dysgenesis

**Figure 7.**
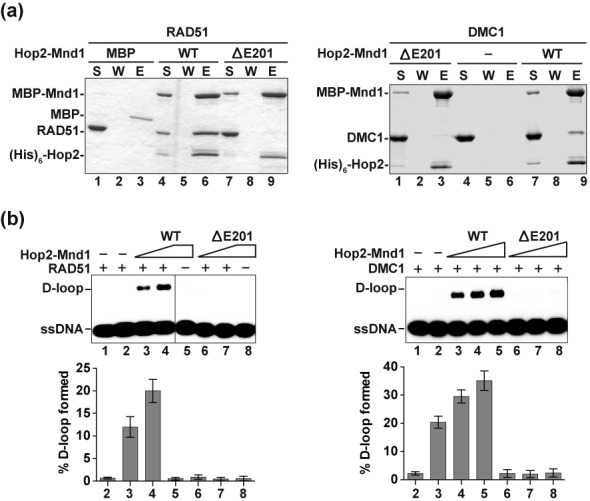
Characterization of Hop2-Mnd1 with the XX-GD HOP2 p.Glu201del (ΔE201) mutation. **(a)** Pulldown assay to test for the interaction of RAD51 (left panel) or DMC1 (right panel) with MBP-tagged Hop2-Mnd1 and Hop2-ΔE201-Mnd1 using amylose resin to capture protein complexes. Analysis was conducted as in Figure 4a. **(b)** Hop2-Mnd1 and Hop2-ΔE201-Mnd1 (90 and 180 nM for RAD51; 60, 90 and 120 nM for DMC1) were tested for their ability to promote D-loop formation by RAD51 (left panel) or DMC1 (right panel). The mean values ± s.d. from three independent experiments were plotted.

It was reported recently that a 3 bp deletion in the human *HOP2* gene, which causes the loss of E201 (p.Glu201del) in the encoded protein, leads to XX gonadal dysgenesis (XX-GD), characterized by streak ovaries and the resultant lack of secondary sex characteristics (20). Since p.Glu201del is located in the RAD51/DMC1 interaction domain of Hop2 (Figure 5b), we wished to examine the effect of this mutation on the physical and functional interactions of Hop2-Mnd1 with DMC1 and RAD51. For this purpose, we expressed and purified Hop2-Mnd1 that harbors the Hop2-ΔE201 mutant for biochemical testing. Importantly, our analyses revealed that Hop2-ΔE201-Mnd1 (i) while being proficient in DNA binding (data not shown and summarized in Table 1) is strongly impaired for interaction with both DMC1 and RAD51 (Figure 7a), and (ii) is devoid of the ability to enhance D-loop formation (Figure 7b) catalyzed by either DMC1 or RAD51.

## DISCUSSION

**Figure 8.**
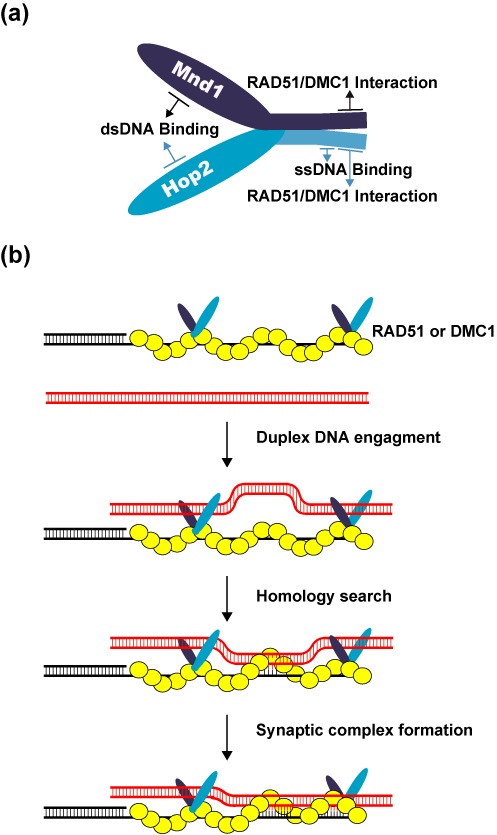
Model for the mechanism of the Hop2-Mnd1-RAD51/DMC1-ssDNA ensemble. **(a)** Cartoon showing the Hop2-Mnd1 complex and functional domains in the protein subunits. **(b)** Our model posits that Hop2–Mnd1 associates with RAD51/DMC1 through an interface contributed by the C-termini of Hop2 and Mnd1, stabilizes the RAD51/DMC1 presynaptic filament via its ssDNA-binding activity within the C-terminus of Hop2 and helps capture duplex DNA by its dsDNA binding functions located in the N-termini of Hop2 and Mnd1. These attributes of Hop2-Mnd1 enhance the efficiency at which a duplex DNA molecule is engaged and sampled for homology by the RAD51/DMC1 presynaptic filament.

Even though the HR role of Hop2-Mnd1 in the budding and fission yeasts is restricted to functional synergy with Dmc1 in meiosis (6,15), there is now compelling evidence that it is also expressed in somatic cells of higher eukaryotes and supports Rad51-mediated HR reactions therein (16,17,27,28). Hop2-Mnd1 is a ‘V’ shaped heterodimer that possesses three distinct DNA binding domains (Figure 8a) (12,29,30). We have shown that ssDNA engagement by the Hop2 C-terminal DNA binding domain helps stabilize the DMC1 presynaptic filament, and that the N-terminal dsDNA binding functions of Hop2-Mnd1 co-operate with the presynaptic filament to mediate synaptic complex assembly (Figure 8b). In this study, we have extended our analysis of Hop2-Mnd1 by testing the functional relevance of its DNA-binding activities in the RAD51-mediated HR reaction. The results show that, as in the case with DMC1, the Hop2 C-terminal DNA binding domain is important for the stabilization of the RAD51 presynaptic filament, and that the N-terminal DNA binding domains of Hop2-Mnd1 work in conjunction with the presynaptic filament to assemble the synaptic complex (Figure 8b). Therefore, the DNA-binding activities of Hop2-Mnd1 fulfill the same functional roles in the HR reaction catalyzed by either DMC1 or RAD51 (Figure 8b).

We have also endeavored to dissect the manner in which Hop2-Mnd1 physically engages Rad51 and Dmc1, and to ascertain the functional significance of these protein complexes. Interestingly, our results reveal that the interaction of Hop2-Mnd1 with RAD51 is enhanced by ATP. In contrast, the association of Hop2-Mnd1 with DMC1 is insensitive to ATP. Since Hop2-Mnd1 is not expected to bind ATP, we postulate that ATP affects a conformational change in RAD51 that is conducive for its interaction with Hop2-Mnd1. Previous work has implicated Mnd1 in Rad51 and Dmc1 interactions (12,13). Importantly, we have shown here that Hop2 also contributes to the association of Hop2-Mnd1 with RAD51. We have localized the RAD51 interaction domains to the C-termini of Hop2 and Mnd1 and have generated variants of these proteins that harbor point mutations in the delineated domains. These mutants are not only attenuated for RAD51 interaction, but are also impaired in the ability to enhance the RAD51-mediated D-loop reaction. Importantly, the various Hop2 and Mnd1 mutants are similarly compromised for physical and functional interactions with DMC1. These results allow us to deduce that Hop2-Mnd1 employs the same protein domains to associate with DMC1 and RAD51. We note that the manner in which Hop2-Mnd1 interacts with DMC1 and RAD51 represents a marked deviation from how distinct domains in other HR factors, e.g. BRCA2 and RAD51AP1, mediate complex formation with the recombinase proteins (31–33).

The experimental frameworks presented herein and elsewhere (12) should be valuable for understanding the biochemical effects of pathological mutations, such as those associated with early onset breast and ovarian cancer risk (18,19) or with XX-GD (20), in the Hop2-Mnd1 complex. In fact, employing the systems that we have devised, we have provided evidence that the XX-GD *HOP2* p.Glu201del mutation (20) adversely affects physical and functional interactions of Hop2-Mnd1 with DMC1 and RAD51. In this regard, even though it has been speculated that the XX-GD *HOP2* mutation impairs hormonal signaling via a transcriptional mechanism (20), our results highlight the possibility that pathological changes in this disease may stem from chromosome damage repair in mitotic and/or meiotic tissues. Such a premise is consistent with the similarity of XX-GD patients and female mice lacking either Hop2 or Mnd1, wherein defective gonadal development (much reduced size of uterus, tubulo-stromal hyperplasia and a lack of follicles) is seen (34,35).

## SUPPLEMENTARY DATA

Supplementary Data are available at NAR Online.

SUPPLEMENTARY DATA

## References

[1] San Filippo J., Sung P., Klein H. (2008). Mechanism of eukaryotic homologous recombination. Annu. Rev. Biochem..

[2] Gerton J.L., Hawley R.S. (2005). Homologous chromosome interactions in meiosis: diversity amidst conservation. Nat. Rev. Genet..

[3] Neale M.J., Keeney S (2006). Clarifying the mechanics by formation of DNA double-strand breaks: mechanism and regulation of DNAstrand exchange in meiotic recombination. Nature.

[4] Bishop D.K., Park D., Xu L., Kleckner N. (1992). DMC1: a meiosis-specific yeast homolog of E. coli recA required for recombination, synaptonemal complex formation, and cell cycle progression. Cell.

[5] Symington L.S. (2002). Role of RAD52 epistasis group genes in homologous recombination and double-strand break repair. Microbiol. Mol. Biol. Rev..

[6] Chen Y.K., Leng C.H., Olivares H., Lee M.H., Chang Y.C., Kung W.M., Ti S.C., Lo Y.H., Wang A.H., Chang C.S. (2004). Heterodimeric complexes of Hop2 and Mnd1 function with Dmc1 to promote meiotic homolog juxtaposition and strand assimilation. Proc. Natl Acad. Sci. U.S.A..

[7] Petukhova G.V., Pezza R.J., Vanevski F., Ploquin M., Masson J.Y., Camerini-Otero R.D. (2005). The Hop2 and Mnd1 proteins act in concert with Rad51 and Dmc1 in meiotic recombination. Nat. Struct. Mol. Biol..

[8] Enomoto R., Kinebuchi T., Sato M., Yagi H., Kurumizaka H., Yokoyama S. (2006). Stimulation of DNA strand exchange by the human TBPIP/Hop2-Mnd1 complex. J. Biol. Chem..

[9] Ploquin M., Petukhova G.V., Morneau D., Dery U., Bransi A., Stasiak A., Camerini-Otero R.D., Masson J.Y. (2007). Stimulation of fission yeast and mouse Hop2-Mnd1 of the Dmc1 and Rad51 recombinases. Nucleic Acids Res..

[10] Uanschou C., Ronceret A., Von Harder M., De Muyt A., Vezon D., Pereira L., Chelysheva L., Kobayashi W., Kurumizaka H., Schlogelhofer P. (2013). Sufficient amounts of functional HOP2/MND1 complex promote interhomolog DNA repair but are dispensable for intersister DNA repair during meiosis in Arabidopsis. Plant Cell.

[11] Pezza R.J., Voloshin O.N., Vanevski F., Camerini-Otero R.D. (2007). Hop2/Mnd1 acts on two critical steps in Dmc1-promoted homologous pairing. Genes Dev..

[12] Zhao W., Saro D., Hammel M., Kwon Y., Xu Y., Rambo R.P., Williams G.J., Chi P., Lu L., Pezza R.J. (2014). Mechanistic insights into the role of Hop2-Mnd1 in meiotic homologous DNA pairing. Nucleic Acids Res..

[13] Chi P., San Filippo J., Sehorn M.G., Petukhova G.V., Sung P. (2007). Bipartite stimulatory action of the Hop2-Mnd1 complex on the Rad51 recombinase. Genes Dev..

[14] Bugreev D.V., Huang F., Mazina O.M., Pezza R.J., Voloshin O.N., Camerini-Otero R.D., Mazin A.V. (2014). HOP2-MND1 modulates RAD51 binding to nucleotides and DNA. Nat. Commun..

[15] Chan Y.L., Brown M.S., Qin D., Handa N., Bishop D.K. (2014). The 3rd exon of the budding yeast meiotic recombination gene HOP2 is required for calcium-dependent and recombinase Dmc1-specific stimulation of homologous strand assimilation. J. Biol. Chem..

[16] Domenichini S., Raynaud C., Ni D.A., Henry Y., Bergounioux C. (2006). Atmnd1-delta1 is sensitive to gamma-irradiation and defective in meiotic DNA repair. DNA Repair (Amst.).

[17] Cho N.W., Dilley R.L., Lampson M.A., Greenberg R.A. (2014). Interchromosomal homology searches drive directional ALT telomere movement and synapsis. Cell.

[18] Peng M., Bakker J.L., Dicioccio R.A., Gille J.J., Zhao H., Odunsi K., Sucheston L., Jaafar L., Mivechi N.F., Waisfisz Q. (2013). Inactivating mutations in GT198 in familial and early-onset breast and ovarian cancers. Genes Cancer.

[19] Peng M., Yang Z., Zhang H., Jaafar L., Wang G., Liu M., Flores-Rozas H., Xu J., Mivechi N.F., Ko L. (2013). GT198 splice variants display dominant-negative activities and are induced by inactivating mutations. Genes Cancer.

[20] Zangen D., Kaufman Y., Zeligson S., Perlberg S., Fridman H., Kanaan M., Abdulhadi-Atwan M., Abu Libdeh A., Gussow A., Kisslov I. (2011). XX ovarian dysgenesis is caused by a PSMC3IP/HOP2 mutation that abolishes coactivation of estrogen-driven transcription. Am. J. Hum. Genet..

[21] Sigurdsson S., Trujillo K., Song B., Stratton S., Sung P. (2001). Basis for avid homologous DNA strand exchange by human Rad51 and RPA. J. Biol. Chem..

[22] Sehorn M.G., Sigurdsson S., Bussen W., Unger V.M., Sung P. (2004). Human meiotic recombinase Dmc1 promotes ATP-dependent homologous DNA strand exchange. Nature.

[23] Chi P., Van Komen S., Sehorn M.G., Sigurdsson S., Sung P. (2006). Roles of ATP binding and ATP hydrolysis in human Rad51 recombinase function. DNA Repair (Amst.).

[24] Bugreev D.V., Mazin A.V. (2004). Ca2+ activates human homologous recombination protein Rad51 by modulating its ATPase activity. Proc. Natl Acad. Sci. U.S.A..

[25] Ferrin L.J., Camerini-Otero R.D. (1991). Selective cleavage of human DNA: RecA-assisted restriction endonuclease (RARE) cleavage. Science.

[26] Namsaraev E.A., Berg P. (1998). Interaction of Rad51 with ATP and Mg2 +induces a conformational change in Rad51. Biochemistry.

[27] Ko L., Cardona G.R., Henrion-Caude A., Chin W.W. (2002). Identification and characterization of a tissue-specific coactivator, GT198, that interacts with the DNA-binding domains of nuclear receptors. Mol. Cell. Biol..

[28] Zierhut C., Berlinger M., Rupp C., Shinohara A., Klein F. (2004). Mnd1 is required for meiotic interhomolog repair. Curr. Biol..

[29] Pezza R.J., Petukhova G.V., Ghirlando R., Camerini-Otero R.D. (2006). Molecular activities of meiosis-specific proteins Hop2, Mnd1, and the Hop2-Mnd1 complex. J. Biol. Chem..

[30] Moktan H., Guiraldelli M.F., Eyster C.A., Zhao W., Lee C.Y., Mather T., Camerini-Otero R.D., Sung P., Zhou D.H., Pezza R.J. (2014). Solution structure and DNA-binding properties of the winged helix domain of the meiotic recombination HOP2 protein. J. Biol. Chem..

[31] Thorslund T., Esashi F., West S.C. (2007). Interactions between human BRCA2 protein and the meiosis-specific recombinase DMC1. EMBO J..

[32] Dray E., Dunlop M.H., Kauppi L., San Filippo J., Wiese C., Tsai M.S., Begovic S., Schild D., Jasin M., Keeney S. (2011). Molecular basis for enhancement of the meiotic DMC1 recombinase by RAD51 associated protein 1 (RAD51AP1). Proc. Natl Acad. Sci. U.S.A..

[33] Dunlop M.H., Dray E., Zhao W., Tsai M.S., Wiese C., Schild D., Sung P. (2011). RAD51-associated protein 1 (RAD51AP1) interacts with the meiotic recombinase DMC1 through a conserved motif. J. Biol. Chem..

[34] Petukhova G.V., Romanienko P.J., Camerini-Otero R.D. (2003). The Hop2 protein has a direct role in promoting interhomolog interactions during mouse meiosis. Dev. Cell.

[35] Pezza R.J., Voloshin O.N., Volodin A.A., Boateng K.A., Bellani M.A., Mazin A.V., Camerini-Otero R.D. (2014). The dual role of HOP2 in mammalian meiotic homologous recombination. Nucleic Acids Res..

