# Supplementary material for: Significance of ligand interactions involving Hop2-Mnd1 and the RAD51 and DMC1 recombinases in homologous DNA repair and XX ovarian dysgenesis
Source: Nucleic Acids Res. 2015 Mar 27;43(8):4055–66. doi: 10.1093/nar/gkv259 (PMC4417169; doi:10.1093/nar/gkv259)
Supplement: SUPPLEMENTARY DATA [file supp_43_8_4055__index.html]

Significance of ligand interactions involving Hop2-Mnd1 and the RAD51 and DMC1 recombinases in homologous DNA repair and XX ovarian dysgenesis — SUPPLEMENTARY DATA 

# Significance of ligand interactions involving Hop2-Mnd1 and the RAD51 and DMC1 recombinases in homologous DNA repair and XX ovarian dysgenesis

## SUPPLEMENTARY DATA

**Files in this Data Supplement:**

- SUPPLEMENTARY DATA
