# Supplementary material for: Significance of ligand interactions involving Hop2-Mnd1 and the RAD51 and DMC1 recombinases in homologous DNA repair and XX ovarian dysgenesis
Source: Nucleic Acids Res. 2015 Mar 27;43(8):4055–66. doi: 10.1093/nar/gkv259 (PMC4417169; doi:10.1093/nar/gkv259)
Supplement: SUPPLEMENTARY DATA [file supp_gkv259_nar-00228-m-2015-File010.pdf]

Supporting information (including 4 supplementary figures and legends)

Figure S1

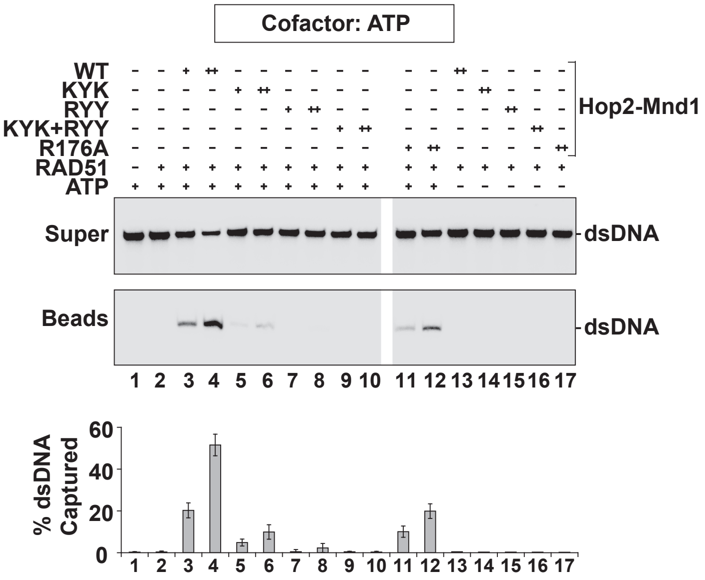

Supplementary Figure S1. Role of the N-terminal DNA binding domains of Hop2-Mnd1 in duplex DNA capture with ATP as nucleotide co-factor.

Wild type (WT) and mutant variants of the Hop2-Mnd1 complex (0.6 or 1.2 μM) were tested for their ability to promote duplex capture with ATP as the nucleotide cofactor. The mean values ± s.d. from three independent experiments were plotted.

Figure S2

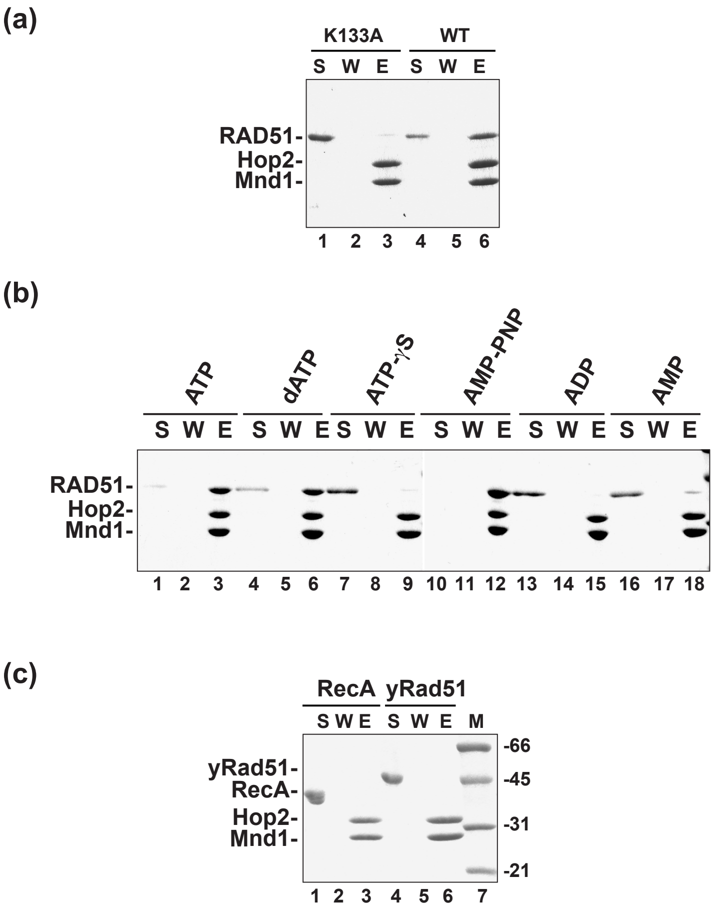

Supplementary Figure S2. Nucleotide cofactor dependence and specificity of the interaction between RAD51 and Hop2-Mnd1.

(a) Pulldown assay to test for the interaction of RAD51 or RAD51(K133A) with $(His)_6$-tagged Hop2-Mnd1 using Ni^{2+}-NTA resin to capture protein complexes. Analysis was conducted as in Figure 4a.

(b) Pulldown assay to test for the interaction of RAD51 with Hop2-Mnd1 as in (a) under various conditions with different nucleotide cofactor as indicated.

(c) Pulldown assay to test for the interaction of Hop2-Mnd1 with RecA from *E. coli.* or Rad51 from *S. cerevisiae* as in (a).

Figure S3

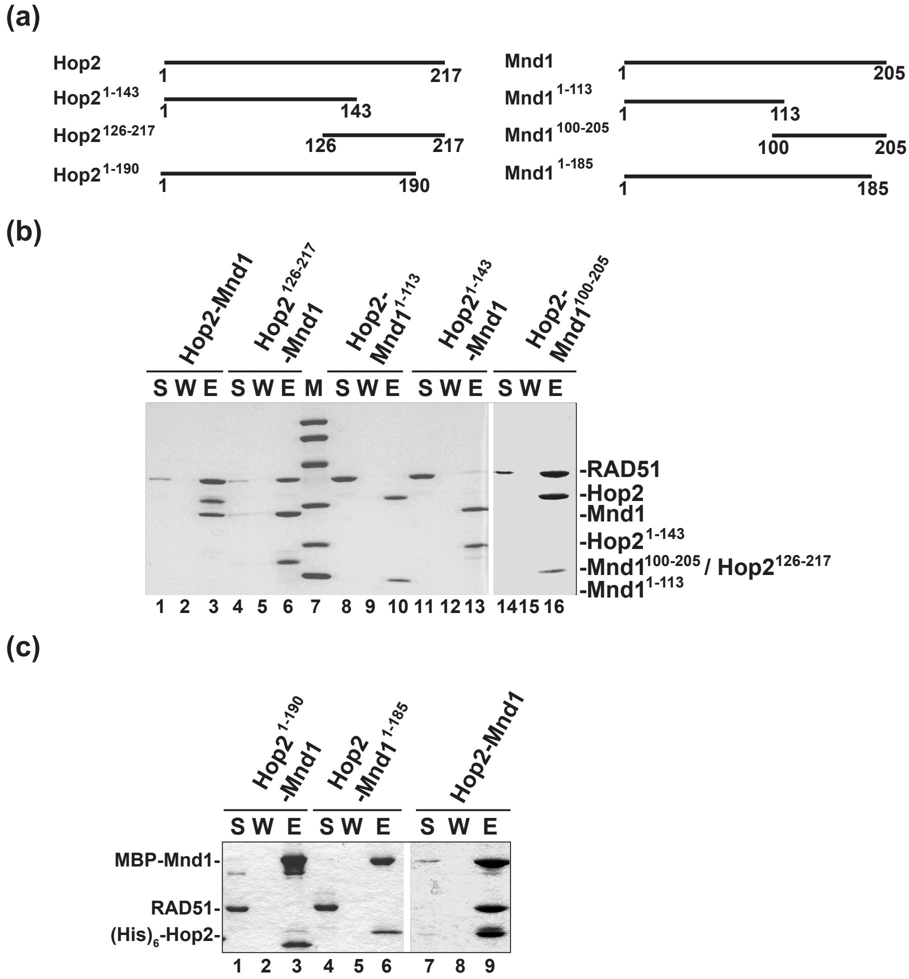

Supplementary Figure S3. The C-termini of Hop2 and Mnd1 are required for interaction of Hop2-Mnd1 with RAD51.

(a) Summary of the Hop2-Mnd1 truncation mutants.

(b) Pulldown assays to test for the interaction of RAD51 with $(His)_6$-tagged truncation mutants of Hop2-Mnd1. Analysis was conducted as in Figure 4a.

(c) Pulldown assay to test for the interaction of RAD51 with $(His)_6$-tagged Hop2-Mnd1 and its truncated mutants (Hop2-Mnd1$^{1-185}$ and Hop2$^{1-190}$-Mnd1) as in (b).

Figure S4

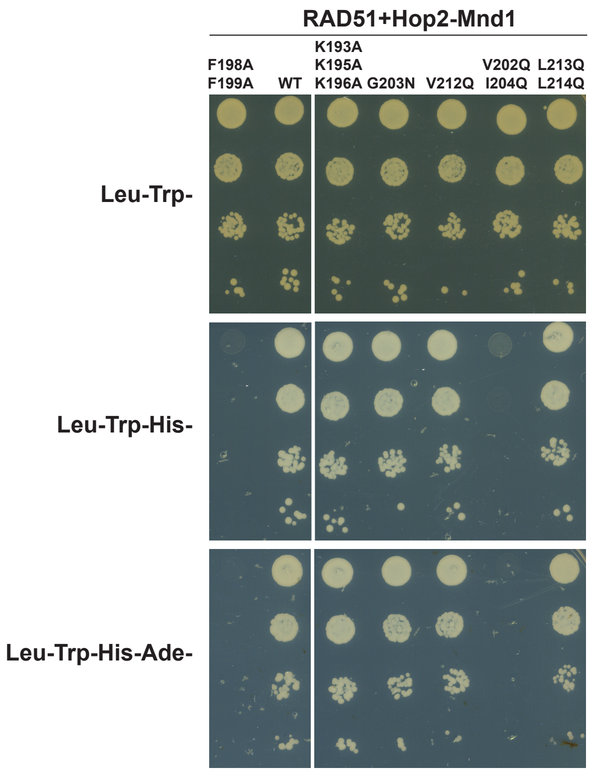

Supplementary Figure S4. Testing of Hop2 mutants in the yeast three-hybrid analysis.

Yeast three hybrid analysis to test the interaction of RAD51 with Hop2-Mnd1 and its various mutants as indicated.
